# Association Between Heavy Metal Exposure and Central Nervous System Tumors: A Case-Control Study Using Single and Multi-Metal Models

**DOI:** 10.3390/toxics13020092

**Published:** 2025-01-26

**Authors:** Sen Luo, Haixia Wu, Fang Xiao, Tianwen Yang, Wei Wang, Hang Du, Peng Su

**Affiliations:** 1 Department of Occupational and Environmental Health, School of Public Health, Chongqing Medical University, Chongqing 400016, China; l384811869@outlook.com; 2 Department of Nursing, The First Affiliated Hospital of Chongqing Medical University, Chongqing 400010, China; 18716676082@139.com; 3 Department of Orthopedics, Sichuan Provincial People’s Hospital, Chengdu 610072, China; 15828573672@163.com; 4 Chongqing Key Laboratory of Prevention and Treatment for Occupational Diseases and Poisoning, The First Affiliated Hospital of Chongqing Medical and Pharmaceutical College, Chongqing 400060, China; ytw2079999@163.com (T.Y.); cqmuwangwei@163.com (W.W.)

**Keywords:** heavy metals, central nervous system, tumors, BKMR analysis

## Abstract

(1) Background: Neoplasms of the central nervous system (CNS) encompass a cluster of malignant diseases originating from tissues or structures within the CNS. Environmental factors, including heavy metals, may contribute to their development. Therefore, this research was to investigate the association between heavy metal exposure and CNS tumor susceptibility using single and muti-metal models. (2) Methods: 63 CNS tumor patients and 71 controls were included. Urine samples from the CNS tumor patients and controls were analyzed for 47 metals using inductively coupled plasma-mass spectrometry in this study. Statistical analyses included conditional Wilcoxon rank-sum tests, logistic regression, Least Absolute Shrinkage and Selection Operator (LASSO) regression, and Bayesian Kernel Machine Regression (BKMR). (3) Results: In the single metal model, higher levels of seventeen metals might be associated with a lower incidence of CNS tumor, while higher exposure levels of five metals are associated with a higher incidence of tumor. LASSO regression selected nine metals for further BKMR analysis. The joint effects showed decreased tumor risk with increased metal mixture concentration. The level of the metals Ge, As, Rb, Zr, and Sn may be related to the incidence of meningiomas and gliomas. (4) Conclusions: This study explored the association between various metals and CNS tumors, providing ideas for future prospective cohort studies and laboratory studies, and providing a foundation for new ideas in the prevention and treatment of CNS tumors.

## 1. Introduction

Tumors of the CNS refer to a group of benign and malignant diseases originating from CNS tissues. The incidence of CNS tumors has been increasing over the years. Between 1990 and 2016, the global number of CNS tumor cases reached 330,000, leading to 227,000 deaths. The age-standardized incidence rate of CNS cancers worldwide increased by 17.3% [1], with a similar trend observed in China. Malignant CNS tumors are associated with a discouraging prognosis as the 5-year relative survival rate after diagnosis of malignant brain and other CNS tumors is 23.5%, compared to 82.4% for non-malignant brain and other CNS tumors [2]. The occurrence and development of CNS tumors may be associated with various factors, such as brain CT scans, viral infections, trauma, and exposure to magnetic fields.

Research into the association between heavy metals and CNS tumors remains limited. Lead (Pb) is considered a potential carcinogen for the CNS [3,4], and elevated levels of blood Pb have been linked to an increased risk of CNS tumors. Moreover, occupational factors associated with Pb exposure also significantly increase the risk of cancer [5]. However, it is worth noting that some of the literature suggests that levels of Pb may not increase the risk of CNS tumors [6,7,8]. Additionally, some literature mentions that the concentrations of heavy metals, such as arsenic (As), thorium (Th), lanthanum (La), lutetium (Lu), cerium (Ce), and gadolinium (Gd) are higher in the patients with brain tumors compared to samples from healthy populations [9]. Another article employed inductively coupled plasma-mass spectrometry (ICP-MS) to detect the blood concentrations of 47 elements in six brain tumor patients and twenty-one healthy subjects, and found that the median levels of cadmium (Cd), Pb, and nickel (Ni) in the blood of the brain tumor patients were higher than the reference values. In addition, significantly higher concentrations of Gd and tantalum (Ta) were detected in those cases. Additionally, distinct differences were observed between tumor and non-tumor brain tissues, as well as characteristic differences in the presence of inorganic elements between tissues from primary tumors and those from brain metastases [10].

Metal biomarkers, primarily including urine, blood, hair, etc., serve as crucial indicators for assessing metal exposure and estimating internal doses. They integrate various sources of exposure, including air, water, and food. Urine contains the most utilized biomarker for assessing metal exposure, and reflects long-term exposure situations [11]. Previous studies have mainly focused on the detrimental effects of individual metals on the CNS, while fewer studies focused on the potential effects of exposure to multiple metals, and they cannot explain the potential multicollinearity between two or more metals. Bayesian Kernel Machine Regression (BKMR) is a pivotal approach for quantifying the health effects of complex mixtures of pollutants [12]. The application of the BKMR model has proven instrumental in studying the relationship between urinary heavy metals and diseases [13].

In this study, we collected 63 cases of CNS tumor patients and then matched them with 71 individuals without benign or malignant CNS tumors, based on age and gender. Urine samples were analyzed, using ICP-MS to detect metals, aiming to explore the association between various metal elements and the occurrence of CNS tumors, thus providing new evidence for the prevention and treatment of CNS tumors in the future.

## 2. Materials and Methods

### 2.1. Study Population

The specimens from patients afflicted with CNS neoplasms utilized in this experiment were collected from the Department of Oncology of the First Affiliated Hospital of Chongqing Medical University, and the control group samples were obtained from the Health Management Center of the same hospital. CNS tumors were collected from the Department of Oncology as the case group, and all cases were diagnosed by skilled clinicians, underwent tumor resection, and subsequently underwent pathological diagnosis to determine the pathological types of the tumors. Finally, 63 patients with CNS tumors and 71 controls were identified based on a comprehensive analysis of their clinical records and laboratory investigations. All participants in this study voluntarily participated in the survey and signed an informed consent form. In addition, this study was approved by the Ethics Committee of the First Affiliated Hospital of Chongqing Medical University.

### 2.2. Urinary Metals Measurement

Morning urine specimens from cases and controls were collected using uncontaminated containers and stored at −80 °C for further analysis. A 65% nitric acid solution was diluted to 1% (V/V) nitric acid using ultrapure water (resistivity 18.2 MΩ·cm at 25 °C). Metal concentrations were determined by semi-quantitative ICP-MS (PerkinElmer, MA, USA). Prior to testing, frozen urine was thawed at room temperature. Then, 0.2 mL of urine was added to a 10 mL polypropylene centrifuge tube and diluted with 3.8 mL of 1% (V/V) nitric acid. The urine was then centrifuged at 3000 rpm for 10 min at room temperature, followed by machine testing. The workstation was pre-set with semi-quantitative factors for each element prior to testing, and the instrument was calibrated by measuring a tuning solution containing a known concentration (1 μg/mL of beryllium (Be), Ce, iron (Fe), Indium (ln), lithium (Li), magnesium (Mg), Pb, uranium (U)). The instrument was calibrated to automatically perform the semi-quantitative factor corrections based on the data from the measurements and the concentrations of the known elements. Values below the limit of detection (LOD) were replaced with a value of LOD/√2 [14].

Calculation of LOD: Prepare 10 portions of 1% (V/V) nitric acid solution and use ICP-MS to detect each metal concentration in the 1% (V/V) nitric acid solution. Calculate the standard deviation (SD) of each metal based on the results from the 10 measurements.

LOD_metal_ = 3SD_metal_ (1)

There, LOD_metal_ refers to the LOD of each metal detected by ICP-MS; SD_metal_ refers to the SD calculated from each metal concentrations detected in 10 portions of the 1% (V/V) nitric acid solution.

Concentration Calculation: Use 3 portions of 1% (V/V) nitric acid solution for on-line ICP-MS to detect the concentration of metal elements, take the average value as the blank control.

C_metal_ = (C_Sample_ − C_blank_) (2)

There, C_metal_ refers to the final concentration of each metal in each sample; C_Sample_ refers to the test result of each metal in each sample; C_blank_ refers to the result of blank control.

### 2.3. Statistical Analysis

The collated data were analyzed in this study using R4.3.2, SPSS26 analysis software. This study calculates the tumor volume based on the descriptions of tumor size in MRI and CT reports. The tumors are grouped using the quartile method, according to type and volume of tumor. The CNS tumors in this study were classified according to the WHO Classification of Tumors, 5th Edition. The routine blood and urine results from the case and control groups were analyzed using the *t*-test if the data conformed to normal distribution, the Wilcoxon signed-rank test if not, and the chi-square test for qualitative data. The detection rate of metals in urine was analyzed using the chi-square test. Urine metal concentrations were expressed as quartiles of continuous variables. The Wilcoxon signed-rank test was used to compare urine metal concentrations between groups, then adjusted by the false discovery rate (FDR). The chi-square test was used to compare distributional differences between demographics. Among the single metal models, we used logistic regression models to analyze the association between the different exposure levels of metal elements and the CNS tumors, with the current address as a confounder. Odds ratios (ORs) and 95% confidence intervals were calculated for the association between the level of exposure to metals and the risk of CNS tumors, grouped according to quartiles of urinary concentrations of the metals, with those below P25 as the reference, the *p*-value was adjusted by FDR.

Subsequently, Spearman rank correlation analysis was used to evaluate the correlation between 41 metals pairwise. This study used the Least Absolute Shrinkage and Selection Operator (LASSO) regression for metal screening in the multi-metal model, constructed a penalty function λ, and after bringing the largest λ within one standard deviation of the mean error into the regression model, finally screened multiple metals for inclusion in the multi-metal analytical model. For the polymetallic analytical model, Bayesian Kernel Machine Regression (BKMR) was used in this study to further explore the mixing effects among several metal elements. The analysis method of BKMR referred to the articles from Bobb et al. (2018) [15], Zhang et al. (2019) [16] and Song et al. (2023) [17]. The posterior inclusion probability (PIP) was included in the BKMR analysis to quantify the uncertainty regarding whether each variable should be included in the model. Higher PIP values (close to 1) indicated greater importance, while lower values (close to 0) suggested lower importance.

BKMR was conducted in several parts. The first part explores the relationship between each metal element and CNS tumor separately, while all other exposures are fixed at a particular percentile. The second part of the BKMR model, which assesses the overall effect of multiple metal exposures, treats all metals as a metal mixture. This is reflected by plotting the trend of the impact of cumulative exposure levels of this metal mixture on disease risk. The third part of the BKMR compares the effect values of individual metal exposure estimates in the effect plots when the exposure concentrations of all other metals are at different percentiles. The fourth section involves a bivariate model, where the effect plot of one metal on the risk of CNS tumors is shown at various percentiles of another metal. In this case, the concentrations of other metals are fixed at a specific percentile.

## 3. Results

### 3.1. Characteristics of Participants

A total of 134 participants, comprising 63 patients with CNS tumors and 71 controls, were included in this study. The mean age of the CNS tumor patient group was 48.7 ± 15.4 years, and the mean age of the control group was 47.3 ± 11.6 years. The case and control groups were stratified into three age categories in this study, namely, under 40 years old, 40–60 years old, and over 60 years old. The chi-square test showed that the difference between the ages of the two groups had a *p*-value of 0.746. From the gender point of view, there were a total of 73 males and 61 females, with a male-to-female ratio of 1.2:1, with a *p*-value of 0.911 for the gender difference between the two groups. The vast majority of the data received were of Han Chinese ethnicity, with two people of other ethnicities in the control group and one in the case group. In terms of the present address, the results showed that there was a statistically significant difference between the current addresses of the control group and the case group. There was no significant difference between smoking and underlying diseases, including diabetes and hypertension; coronary heart disease was not included in the statistics due to the lack of a complete clinical diagnosis (Table 1).

### 3.2. Other Tests in Cases and Controls Group

Tumor volumes were calculated based on measurements from computed tomography (CT) and magnetic resonance imaging (MRI) reports, with cases subsequently stratified into quartiles for analysis. Analysis revealed a uniform distribution of CNS tumor size across all four quartile groups (Table S1). Hematological analysis demonstrated significant differences between the case and control groups. The case group exhibited elevated levels of total protein, albumin, globulin, albumin-to-globulin ratio, leukocyte counts, neutrophil counts and percentages, and monocyte counts and percentages. Conversely, the case group showed decreased values in red blood cell count, hemoglobin content, hematocrit, mean hemoglobin content, mean hemoglobin concentration, platelet distribution width, lymphocyte measurements (both absolute and percentage), eosinophil measurements (both absolute and percentage), and basophil percentages. Biochemical parameters including total bilirubin, direct bilirubin, gamma-glutamyl transferase, creatinine, uric acid, and total cholesterol were significantly elevated in the control group. Urinalysis revealed statistically significant differences between control and case groups in urine pH, protein characterization, and conductivity measurements (Tables S2 and S3).

### 3.3. Comparison of Detection Limits for 47 Urinary Metals

In this study, ICP-MS was used for the determination of urinary metallic elements, and the chi-square test was used to statistically analyze the detection rate between the control group and the case group (Table S4).

### 3.4. Urinary Metals Concentrations

**Figure 1:**
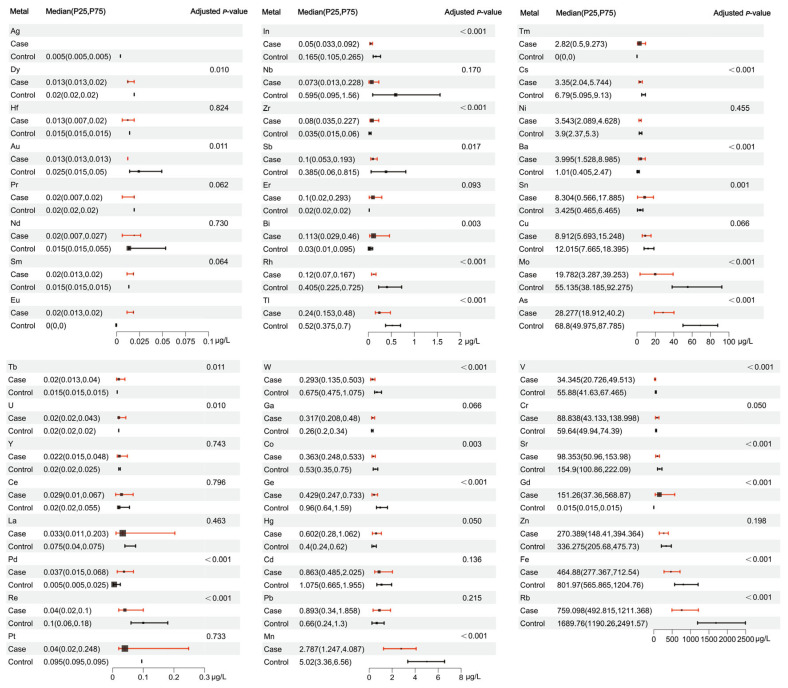
Exposure levels of 47 metals in case group and control group. The first column of the figure represents metals and their groupings, the second and third columns represent the median, P25, and P75 of metal concentrations, and the last column shows the *p*-value from the Wilcoxon rank-sum test.

The Wilcoxon rank sum test was used in this study. A statistically significant distinction was observed in the concentration of urinary metals between the case group and the control group for 26 metals. The results are shown in Table S5 and Figure 1.

### 3.5. Correlation of Urinary Metals with Risk of CNS Tumors in Single Metal Model

We included 32 metals in the single metal model. The metals vanadium (V), manganese (Mn), chromium (Cr), iron (Fe), cobalt (Co), germanium (Ge), As, rubidium (Rb), strontium (Sr), molybdenum (Mo), rhodium (Rh), In, antimony (Sb), cesium (Cs), tungsten (W), rhenium (Re), and thallium (Tl) are negatively correlated with CNS tumors, while metals zirconium (Zr), tin (Sn), barium (Ba), bismuth (Bi), and U are positively correlated. In addition, the *p* value of metal copper (Cu) was 0.055, but in Quartile 3 and Quartile 4, the OR and 95% CI were 0.268 (0.084, 0.858) and 0.193 (0.058, 0.643), respectively, showed a negative correlation between CNS tumors and metal Cu (Table 2).

### 3.6. Multiple Metals Exposure Model for CNS Tumors

**Figure 2:**
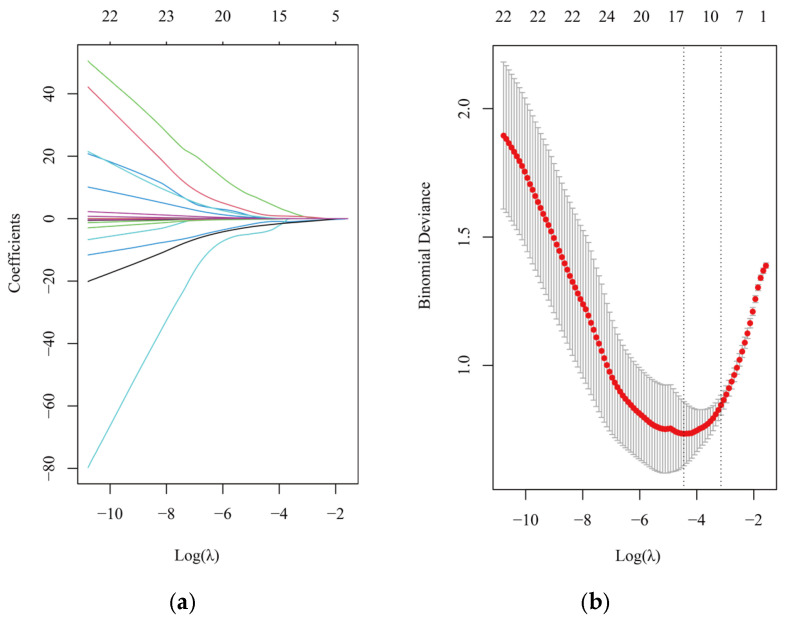
The multiple-metals model using LASSO regression method. (**a**) The prediction error of the LASSO regression model in function of the penalty parameter (log λ). (**b**) The LASSO solution path, with the coefficient profiles for 27 urine metals as a function of the penalty parameter (log λ).

Spearman rank correlation was used to test the correlation between 41 metals (Figure S1). When the coefficient is greater than 0.7, it indicates that their sources may be the same. There are connections between various metals. Afterwards, a multi-metal model was constructed in this study, and 27 metals were screened for using LASSO regression after excluding some metals that were below the detection limit. Cr, Ga, Ge, As, Rb, Zr, Sn, Sb, and Pb were selected for inclusion in the multi-metal model in Figure 2.

### 3.7. Bayesian Kernel Machine Regression (BKMR) Analyses

**Figure 3:**
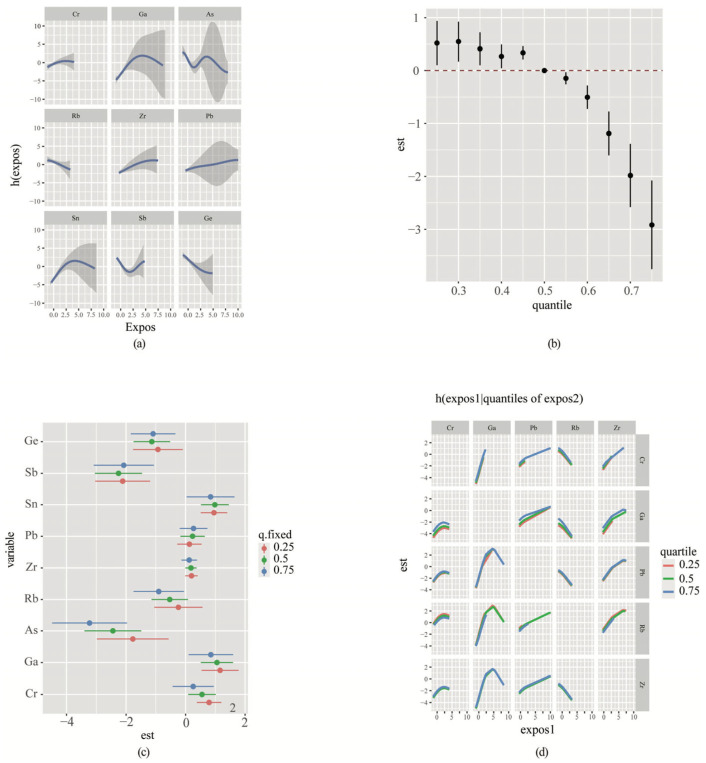
The BKMR models of each metal and mixed exposure effects on tumors of the CNS. (**a**) Univariate exposure–response functions of each metal, along with their respective 95% confidence intervals (95% CI), while keeping the concentrations of other metals fixed at their medians. (**b**) The overall effects of mixed exposure, with metals fixed at different percentiles compared to their concentrations when they were at their medians (50th). (**c**) The effects of a single exposure when an individual metal was at its 75th percentile, contrasted with the scenario when that exposure is at its 25th percentile. Notably, all other exposures were fixed at 75th. (**d**) The bivariate cross-section effects of the exposure–response function of a single metal, where the second 30 metal was fixed at 25th, 50th, and 75th. This analysis provides insights into the joint effects of these metals at different concentration levels.

Next, we assessed the impact of LASSO regression on polymetallic mix exposure using the BKMR model. The PIP was calculated for each metal (Table S6) to determine their respective importance. The Univariate exposure–response functions were plotted as the levels of all other metals reached their median concentrations (Figure 3a). Notably, Zr and Pb showed positive relationships with the risk of CNS tumors, while Rb and Ge demonstrated negative relationships with the risk of CNS tumors.

The overall correlations of the mixed metals were shown in Figure 3b. Using the median mixture level as a reference value, our model demonstrates the relationship between metal mixture concentrations and the risk of CNS tumors, with 95% confidence intervals represented by the vertical lines. The analysis reveals an inverse association: concentrations above the median exhibit a negative correlation with CNS tumor risk, while concentrations below the median show a positive correlation with CNS tumor risk.

In Figure 3c, the correlation between metal exposure and CNS tumors was illustrated (75th vs. 25th), keeping the other eight metals fixed at different percentiles (25th, 50th, or 75th). Results exhibited that the metals Ga and Sn were positively associated with the risk of CNS tumors, especially when the concentrations of other metals were low (25th). Conversely, Ge, Sb, and As were negatively associated with the risk of CNS tumors and, in the case of As, this was more pronounced at lower concentrations of other metals (25th). Figure 3d demonstrated a potential joint effect of metals on increasing the risk of CNS tumors. The slope of Rb effect curve became steeper at higher concentrations of urinary Ga. Conversely, when urinary Ga concentration increased, the slope of Pb effect curve became flattened. And the slope of Ga effect curve was not significantly changed by changes in Pb and Rb.

### 3.8. Correlation of Urinary Metals with Risk of Different Types of CNS Tumors in the Single Metal Model

Table 3 displays that the high concentrations of the metal Ge (Quartile 4) could be negatively correlated with the risk of glioma. Any concentration of the metal As was negatively associated with the risk of glioma and meningioma., Similarly to As, Rb was also negatively associated with the risk of glioma and meningioma, but the highest concentration group had zero patients in the case group. The metal Zr was positively associated with the risk of gliomas and meningiomas, whereas the metal Sn could be positively associated with the risk of meningioma.

## 4. Discussion

In this study, we aimed to investigate the concentrations of urinary metals and their combined effects on the risk of CNS tumors. The findings indicated that higher levels of some metals (V, Mn, Fe, Co, Ge, As, Rb, Sr, Mo, Rh, In, Sb, Cs, W, Re, and Tl) might be associated with a lower incidence of CNS tumor, while higher exposure levels of other metals (Zr, Sn, Ba, Bi, and U) are associated with a higher incidence of tumor. The BKMR models revealed that Zr and Pb were positively associated with CNS tumors, while urinary Rb and Ge were negatively associated. The bivariate exposure–response analysis revealed complex interactions among metals, demonstrating a synergistic effect between Ga and Rb, while simultaneously showing an antagonistic interaction between Ga and Pb. Our findings indicate differential associations between specific metal exposures and CNS tumor subtypes: elevated concentrations of As and Rb were inversely associated with both glioma and meningioma incidence, whereas increased Zr levels showed positive associations with both tumor types. Furthermore, higher Ge concentrations demonstrated an inverse association specifically with glioma incidence, while elevated Sn levels were positively associated with meningioma incidence.

Our findings demonstrate complex associations between metal concentrations and CNS tumor risk, with elevated levels of specific metals showing differential correlations with tumor incidence. These metal–metal interactions exhibit both synergistic and antagonistic effects, suggesting that the relationship between metal exposure and CNS tumor development is multifaceted. The observed patterns indicate the potential utility of targeted screening protocols for populations with a higher metal burden, while the identified interactions suggest that carefully managed trace element supplementation might serve as a preventive intervention strategy. Further research is necessary to elucidate the underlying biological mechanisms and validate these preliminary findings.

Beyond metal exposure, demographic and geographical factors significantly influence cancer incidence patterns. Gender emerges as a crucial determinant in cancer susceptibility, with distinct patterns attributable to hormonal variations and lifestyle differences between males and females [18]. Specifically, regarding CNS tumors, epidemiological data indicates a higher prevalence of malignant brain tumors in males, while benign meningiomas demonstrate increased frequency among adult females. These gender-specific variations in CNS tumor patterns are further modulated by age and racial factors [19]. Geographical location and environmental exposure also demonstrate significant associations with tumor risk, particularly evident in the elevated neuroblastoma incidence observed at industrial–urban interfaces and in proximity to mines, metal processing facilities, and sewage treatment installations [20]. Furthermore, socioeconomic disparities within nations manifest in differential cancer outcomes, with rural populations experiencing notably lower survival rates compared to their urban counterparts [21].

### 4.1. The Sources of the Metals

Heavy metal pollution has emerged as a pervasive global environmental concern, with multiple anthropogenic sources contributing to increased metal exposure risks. Industrial activities, agricultural practices, and urbanization processes serve as primary pathways for heavy metal contamination. Empirical evidence from various geographical locations demonstrates the severity of this issue. For instance, in San André, Brazil, Ba concentrations in agricultural soil exceed the established standard range of 20–112 mg·kg^−1^, while urban soils exhibit moderate to severe contamination by As, Ba, Cr, Cu, Pb, and Zn, with As, Cr, and V identified as the predominant pollutants [22]. Similarly, in Xingyang City, Henan Province, soil analysis reveals elevated heavy metal content in arable land, with As, Cu, Hg, Pb, and other metals showing concentrations 1.04–1.40 times higher than the background levels recorded in Zhengzhou City, notably featuring significant Cd accumulation from industrial sources [23]. A comprehensive review of 713 studies conducted between 2000 and 2019 on heavy metal contamination in Chinese soils revealed substantial increases in Cd levels, with concentrations doubling in farmland soil and tripling in urban soil, resulting in Cd contamination affecting 33.54% of farmland and 44.65% of urban soil [24]. Occupational exposure represents another significant pathway for heavy metal contamination, as evidenced by elevated levels of Cd, Cr, and Ni in female brick kiln workers [25], while lead and zinc miners demonstrate increased susceptibility to Pb, Cd, Cu, Ni, and As exposure [26].

### 4.2. Ga Concentrations and Health

In this study, the multi-metal model indicates that, with the concentration of other metals fixed, Ga is positively associated with the risk of CNS tumors, particularly when the concentration of other metals is low. The toxicity of gallium metal has been less extensively studied, but Liao’s article demonstrated that lipid peroxidation was increased in workers with elevated urinary Ga levels [27], and that lipid peroxidation plays a pivotal role in primary and metastatic brain tumors [28].

### 4.3. V Concentrations and Health

V concentrations in the control group were significantly higher than that in the case group, and the highest concentration of V was negatively associated with the risk of CNS tumors in the monometallic model. This findings aligns with the previous suggestion that V plays an important role in anti-tumor activity [29]. Although V has been less extensively studied in CNS tumors, its anti-tumor effects were established as early as the mid-20th century, with indirect inhibitory effects on human rhabdomyosarcoma, human lung and prostate cell lines, and tumor-unaltered bronchial epithelial cells [30]. The anti-tumor properties of V may be due to its inhibitory effect on tyrosine phosphatase and tyrosine phosphorylation enzymes [31].

### 4.4. Cr Concentrations and Health

Cr is a recognized carcinogen. Hara et al. (2010) found that exposure to hexavalent Cr increases the risk of brain cancer mortality (SMR = 9.14, Obs = 3, 95% CI 1.81–22.09) [32]. Iaia et al. (2006) and Donato et al. (2016) reported that exposure to hexavalent Cr may increase the risk of death from lung cancer, bladder cancer, pancreatic cancer, myeloid leukemia, kidney cancer, testicular cancer, thyroid cancer, bone cancer [33], and endocrine gland tumors among skin contactors [34]. Epidemiological investigations show a significant correlation between environmental hexavalent Cr exposure and lung cancer mortality, as well as incidence of nasal cavity cancer [35]. Research from Li et al. suggested that exposure to heavy metals, including Cr, may affect the levels of lipids and certain low molecular weight metabolites in the human bloodstream, potentially promoting the development of breast cancer [36]. This study observed Cr levels in the serum of breast cancer patients that were 3.24 times higher than in the controls’. In a study focusing on breast cancer patients, Benderli Cihan et al. (2011) compared the levels of 36 elements in hair between stage III breast cancer patients and healthy female controls. The study found a significant increase in the Cr content of the hair of breast cancer patients compared to the control group (*p* < 0.05) [37], which was consistent with our findings.

The mechanisms of Cr-induced carcinogenesis differ between trivalent (Cr(III)) and hexavalent (Cr(VI)) forms. Trivalent chromium (Cr(III)) can react with biomolecules, such as DNA, but its poor ability to penetrate cell membranes is considered one of the reasons for its low biological and toxicological activity. The entry of trivalent chromium into cells through passive diffusion or phagocytosis is a slow process that has often been overlooked in research [38]. Due to its low membrane permeability, Cr(III) can accumulate inside cells hundreds of times over. Additionally, it can induce morphological changes in membranes, disrupt cellular functions and integrity, and ultimately lead to DNA damage [39]. In contrast, hexavalent chromium (Cr(VI)) cannot directly interact with DNA, and no genotoxic effects are observed [40,41]. However, the reduction of Cr(VI) inside cells produces intermediate species, such as pentavalent chromium (Cr(V)) and tetravalent chromium (Cr(IV)), which appear to cause DNA damage. Oxidative stress reactions generate a series of reactive oxygen species (ROS) after Cr(VI) is reduced, such as organic free radicals. These intermediates can oxidize purine and pyrimidine bases in DNA or directly act on guanine bases in DNA. Due to its higher oxidation potential, guanine is more susceptible to oxidation compared to cytosine, thymine, and adenine, potentially leading to DNA damage [42]. Furthermore, transient chromium species and ROS formation trigger a series of events that alter normal cellular functions, damage nucleic acids, oxidize proteins, cause lipid peroxidation, and promote changes in gene expression. DNA damage induced by oxidative stress includes modification of DNA bases, sites of missing nucleotides, sugar damage, single-strand or double-strand breaks in DNA, DNA–protein crosslinks, and DNA–DNA crosslinks, among other types of damages. These functional impairments underscore their genotoxic nature and their potential role in mutation and carcinogenesis [43].

### 4.5. Pb Concentrations and Health

Our results found that the association between Pb and central nervous system tumors is not significant. Meta-analysis from Ahn et al. (2020) reported that occupational Pb exposure was associated with a higher risk of CNS tumors (OR = 1.11, 95% CI: 0.95–1.29), and studies using blood Pb levels showed a significant increase in risk (OR = 1.67, 95% CI: 1.12–2.49). Specifically analyzing malignant tumors, the risk of CNS tumors associated with Pb exposure increased further (OR = 1.13, 95% CI: 1.04–1.24). Additionally, the authors also found that Pb may be associated with changes in the risk of meningiomas (OR = 1.42, 95% CI: 1.09–1.85) [5]. However, this study did not find the effect of Pb on the risk of meningiomas or other types of CNS tumors, possibly due to the smaller number of meningioma cases.

Pb-induced CNS damage may occur through several mechanisms. Firstly, Pb can substitute calcium ions, cross the blood–brain barrier (BBB) and mimic or activate calcium and protein kinase C (PKC), leading to the alteration of neurobehavior and disruption of the BBB [44]. Pb also affects numerous physiological functions, such as intervening protein synthesis and gene expression. Pb disrupts the activity of Ca2+-ATPase, preventing it from binding with calcium, causing excessive intracellular calcium levels, which exacerbate the effects of high concentrations of glutamate or other excitotoxic neurotransmitters, leading to neuronal apoptosis [45].

Secondly, during inflammation processes, Pb plays a crucial role by influencing the expression of many inflammation-related factors, such as IL-6, TGF-β1, IL-16, IL-18, and IL-10. It also affects the expression and activity of enzymes associated with inflammation, such as COX-2, GSH-1, NOS, and proteases, resulting in neuronal cell death [46].

Lastly, prolonged exposure to Pb during development may impair neurotransmission, affecting calcium-dependent glutamate and GABA release, and inhibiting presynaptic voltage-gated calcium channels [47,48].

### 4.6. As Concentrations and Health

As shows a negative correlation with the risk of CNS tumors, suggesting a potential protective effect, even at lower concentrations. In a multi-metal model, As enhances its effects with increasing concentrations of other metals. Currently, As’s damaging effects on the CNS are well-established. For humans, a study of 10-year-old children in Bangladesh found that exposure to As in drinking water correlated with decreased scores on intelligence function tests, with effects increasing with exposure levels [49]. The research of Calderón et al. has linked increased urinary As concentrations with decreased verbal IQ in Mexico [50]. Studies on adolescents indicated that long-term cumulative As exposure significantly affected pattern memory and attentional switching. In mouse experiments, the ingestion of inorganic As through drinking water by pregnant females affects fetal brain development and postnatal behavior [51]. Additionally, As is classified as a Group 1 carcinogen by the International Agency for Research on Cancer, with carcinogenic effects possibly mediated through abnormal DNA repair, aneuploidy, and other cellular mechanisms [52]. Prolonged exposure to As can lead to skin diseases, such as hyperpigmentation, hypopigmentation, Bowen’s disease, and arsenic keratosis, ultimately progressing to skin cancer [53]. However, limited research has been carried out on As’s impact on CNS tumors, and the result of Cilliers et al. contradicted our results [9]. Future research is needed to elucidate the complex relationship between As exposure with CNS tumor risk.

### 4.7. Other Metal and Health

This study also found that the concentration of the metals Fe and Cu were higher in the control group compared to the case group. Dehnhardt et al. (2008) showed that Cu levels in tumor tissues were significantly lower than in adjacent healthy tissues, despite higher tumor cell density [54]. In Cross et al.’s study, both dietary Fe and total Fe showed statistically significant negative correlation with colorectal cancer risk (dietary Fe RR, 0.75; 95% CI, 0.65–0.87; total Fe RR, 0.75; 95% CI, 0.66–0.86) [55].

It is important to note that tumor occurrence can lead to disruption in Fe metabolism. Cancer cells typically alter their internal Fe metabolism to promote Fe accumulation in breast [56], prostate [57] and ovarian cancer [58], which includes increasing Fe uptake and storage, reducing Fe release, or both simultaneously [56,57,59,60].

Finally, the other metals mentioned in this article have not been discussed due to the limited research available on them.

### 4.8. Multi-Metal and Health

Our analysis revealed complex metal–metal interactions, with Ga demonstrating a synergistic effect with Rb, while exhibiting an antagonistic relationship with Pb. These findings align with the previous research by Lim et al. (2019) [61], which demonstrated a significant nonlinear association between serum As and Zn concentrations and prostate cancer risk. Their study further identified that the combined effects of multiple metals were associated with an increased prostate cancer risk, primarily attributed to As and Zn interactions. However, it is noteworthy that contradictory evidence exists in the literature, as exemplified by a separate investigation into metal exposure and glioma risk which failed to establish a significant correlation between metal exposure and glioma development [62].

## 5. Conclusions

In this study, we investigated the association between metal exposure and CNS tumor risk. Our findings reveal a complex relationship between various urinary metals and CNS tumor risk. Specifically, we observed that high concentrations of the urinary metals Zr, Sn, Ba, Bi, and U are associated with a higher incidence of CNS tumors, while high concentrations of the urinary metals V, Mn, Fe, Co, Ge, As, Rb, Sr, Mo, Rh, In, Sb, Cs, W, Re, and Tl are associated with a lower incidence of CNS tumors. Interestingly, the BKMR model found a synergistic effect of the metal Ga on Rb, while an antagonistic effect of the metal Ga on Pb. Further analysis demonstrated that higher levels of the metals As and Rb might be associated with a lower incidence of glioma and meningioma, while higher exposure levels of the metal Zr are associated with a higher incidence of glioma and meningioma. Conversely, higher levels of the metal Ge might be associated with a lower incidence of glioma, and higher exposure levels of the metal Sn are associated with a higher incidence of meningioma. To validate these findings and elucidate the underlying mechanisms, future research will employ prospective cohort studies and experimental approaches using animal models and cellular systems. Ultimately, this research aims to provide a foundation for the development of novel strategies for the prevention and treatment of CNS tumors.

## Figures and Tables

**Table 1:** The basic demographic characteristics of cases and controls.

		Cases	Controls	*p* Value
		(*n* = 63)	(*n* = 71)	
Age	~40	13	18	
	40~60	33	39	
	60~	15	14	0.746
Gender	Male	34	39	
	Female	29	32	0.911
Nationality	Han Chinese	60	71	1
	Others	1	2	
Residential address	the downtown of Chongqing	21	25	0.010
	Western Chongqing	9	13	
	Northeast Chongqing	13	9	
	Southeast Chongqing	3	4	
	Other provinces	16	2	
Smoking	Yes	7	9	0.946
	No	50	62	
Underlying diseases	Hypertension	10	11	0.367
	Diabetes	4	1	
	Hypertension and diabetes	3	5	
	None	36	54	

**Table 2:** OR (95% CI) for CNS tumor according to quartiles of urine metal exposure in the single metal model.

	Quartile 1	Quartile 2	Quartile 3	Quartile 4	Adjusted *p* Value
V					
Range	≤29.34	~46.98	~63.201	>63.201	
Case/Control	25/12	19/13	14/18	5/27	
OR (95%CI)	1	0.499 (0.159, 1.565)	0.361 (0.118, 1.104)	0.043 (0.01, 0.181)	0.001
Cr					
Range	≤29.34	~46.98	~104.44	>104.44	
Case/Control	18/14	7/25	13/19	25/7	
OR (95%CI)	1	0.223 (0.069, 0.719)	0.462 (0.155, 1.373)	2.889 (0.905, 9.226)	0.001
Mn					
Range	≤2.16	~3.73	~6.18	>6.18	
Case/Control	22/8	19/12	8/24	10/19	
OR (95%CI)	1	0.571 (0.179, 1.817)	0.075 (0.02, 0.275)	0.09 (0.024, 0.341)	<0.001
Fe					
Range	≤353.84	~643.61	~1018.81	>1018.81	
Case/Control	20/11	20/11	11/21	8/23	
OR (95%CI)	1	1.029 (0.338, 3.132)	0.308 (0.101, 0.935)	0.196 (0.06, 0.64)	0.013
Co					
Range	≤0.33	~0.44	~0.65	>0.65	
Case/Control	24/10	14/15	11/23	11/19	
OR (95%CI)	1	0.36 (0.118, 1.095)	0.17 (0.055, 0.521)	0.262 (0.086, 0.801)	0.023
Ni					
Range	≤2.24	~3.78	~5.13	>5.13	
Case/Control	16/16	14/17	16/17	12/19	
OR (95%CI)	1	1.143 (0.391, 3.344)	0.841 (0.291, 2.429)	0.495 (0.163, 1.502)	0.509
Cu					
Range	≤6.66	~10.79	~17.24	>17.24	
Case/Control	18/11	12/17	11/18	9/20	
OR (95%CI)	1	0.421 (0.136, 1.297)	0.268 (0.084, 0.858)	0.193 (0.058, 0.643)	0.055
Zn					
Range	≤170.16	~296.8	~443.04	>443.04	
Case/Control	19/13	15/16	14/19	13/18	
OR (95%CI)	1	0.673 (0.23, 1.974)	0.361 (0.12, 1.082)	0.461 (0.155, 1.369)	0.313
Ga					
Range	≤0.2	~0.28	~0.4	>0.4	
Case/Control	15/18	10/17	15/25	22/7	
OR (95%CI)	1	0.724 (0.245, 2.138)	0.696 (0.261, 1.854)	2.456 (0.772, 7.812)	0.166
Ge					
Range	≤0.33	~0.68	~1.09	>1.09	
Case/Control	23/9	19/13	15/17	2/30	
OR (95%CI)	1	0.969 (0.302, 3.11)	0.348 (0.106, 1.142)	0.005 (0, 0.065)	0.001
As					
Range	≤28.15	~47.17	~72.94	>72.94	
Case/Control	31/2	21/10	6/26	4/28	
OR (95%CI)	1	0.198 (0.038, 1.041)	0.015 (0.002, 0.088)	0.009 (0.001, 0.057)	<0.001
Rb					
Range	≤716.1	~1204.16	~1842.18	>1842.18	
Case/Control	29/3	18/13	7/26	8/23	
OR (95%CI)	1	0.174 (0.041, 0.73)	0.037 (0.008, 0.162)	0.037 (0.008, 0.164)	<0.001
Sr					
Range	≤70.79	~127.81	~191.18	>191.18	
Case/Control	28/6	13/19	13/19	9/23	
OR (95%CI)	1	0.138 (0.041, 0.459)	0.14 (0.042, 0.469)	0.08 (0.023, 0.28)	0.001
Zr					
Range	≤0.02	~0.05	~0.14	>0.14	
Case/Control	3/21	17/6	8/15	19/4	
OR (95%CI)	1	27.743 (5.204, 147.895)	2.995 (0.59, 15.209)	29.864 (5.211, 171.148)	<0.001
Mo					
Range	≤19.08	~38.66	~76.14	>76.14	
Case/Control	30/2	17/15	7/25	9/23	
OR (95%CI)	1	0.079 (0.015, 0.406)	0.022 (0.004, 0.119)	0.025 (0.005, 0.135)	<0.001
Rh					
Range	≤0.1	~0.22	~0.5	>0.5	
Case/Control	18/7	19/6	7/18	1/24	
OR (95%CI)	1	1.093 (0.271, 4.415)	0.1 (0.023, 0.437)	0.005 (0, 0.078)	<0.001
Pd					
Range	≤0.01	~0.03	~0.05	>0.05	
Case/Control	1/10	10/0	11/5	10/0	
OR (95%CI)	1	1	1	1	0.112
Cd					
Range	≤0.58	~1.04	~1.98	>1.98	
Case/Control	21/12	13/16	12/22	16/15	
OR (95%CI)	1	0.425 (0.14, 1.29)	0.247 (0.083, 0.739)	0.662 (0.227, 1.93)	0.100
In					
Range	≤0.05	~0.11	~0.2	>0.2	
Case/Control	17/10	15/10	7/22	1/25	
OR (95%CI)	1	0.797 (0.234, 2.715)	0.105 (0.025, 0.447)	0.011 (0.001, 0.122)	0.001
Sn					
Range	≤0.48	~4.49	~10.22	>10.22	
Case/Control	14/17	9/22	10/22	27/4	
OR (95%CI)	1	0.464 (0.149, 1.44)	0.37 (0.116, 1.184)	6.963 (1.848, 26.241)	0.001
Sb					
Range	≤0.05	~0.13	~0.55	>0.55	
Case/Control	13/16	22/13	21/13	4/27	
OR (95%CI)	1	2.513 (0.834, 7.569)	2.267 (0.741, 6.932)	0.142 (0.035, 0.581)	0.001
Cs					
Range	≤3.08	~5.34	~7.72	>7.72	
Case/Control	30/2	13/19	10/22	9/23	
OR (95%CI)	1	0.057 (0.011, 0.288)	0.042 (0.008, 0.22)	0.027 (0.005, 0.143)	0.001
Ba					
Range	≤0.7	~1.83	~4.69	>4.69	
Case/Control	7/23	14/16	14/16	27/3	
OR (95%CI)	1	2.556 (0.817, 7.994)	2.75 (0.885, 8.55)	21.053 (4.731, 93.684)	0.002
La					
Range	≤0.01	~0.06	~0.12	>0.12	
Case/Control	14/0	12/4	5/11	14/1	
OR (95%CI)	1	1	1	1	0.033
Gd					
Range	≤7.4	~85.97	~337.47	>337.47	
Case/Control	6/13	19/0	19/0	19/0	
OR (95%CI)	1	1	1	1	1
W					
Range	≤0.21	~0.5	~0.89	>0.89	
Case/Control	24/8	19/11	7/25	8/23	
OR (95%CI)	1	0.629 (0.198, 1.995)	0.093 (0.027, 0.32)	0.094 (0.027, 0.33)	<0.001
Re					
Range	≤0.04	~0.08	~0.16	>0.16	
Case/Control	25/10	5/7	12/28	7/20	
OR (95%CI)	1	0.269 (0.061, 1.18)	0.159 (0.054, 0.463)	0.15 (0.045, 0.496)	0.005
Hg					
Range	≤0.27	~0.45	~0.8	>0.8	
Case/Control	10/18	9/20	13/17	15/12	
OR (95%CI)	1	0.597 (0.182, 1.96)	0.915 (0.287, 2.921)	1.665 (0.523, 5.299)	0.427
Tl					
Range	≤0.22	~0.41	~0.62	>0.62	
Case/Control	29/4	14/17	11/22	9/23	
OR (95%CI)	1	0.188 (0.05, 0.704)	0.102 (0.027, 0.39)	0.056 (0.014, 0.22)	0.001
Pb					
Range	≤0.26	~0.76	~1.68	>1.68	
Case/Control	12/18	12/17	11/20	19/10	
OR (95%CI)	1	0.923 (0.308, 2.762)	0.676 (0.226, 2.019)	2.138 (0.696, 6.564)	0.274
Bi					
Range	≤0.01	~0.05	~0.2	>0.2	
Case/Control	2/24	10/9	10/17	15/8	
OR (95%CI)	1	11.781 (1.948, 71.238)	9.347 (1.631, 53.559)	19.818 (3.362, 116.832)	0.017
U					
Range	≤0.02	~0.02	~0.03	>0.03	
Case/Control	30/36	0/0	0/0	20/3	
OR (95%CI)	1			5.289 (1.355, 20.646)	0.024

Metals were calculated in the conditional logistic regression model separately and residential address was tested as confounder. The *p* values were adjusted by FDR.

**Table 3:** OR (95% CI) for 3 different types of CNS tumor according to quartiles of urine metal exposure in the single metal model.

		Quartile 1	Quartile 2	Quartile 3	Quartile 4	Adjusted *p* Value
Gliomas	Cr					
	Range	≤29.34	~46.98	~104.44	>104.44	
	Case/Control	8/14	5/25	7/19	8/7	
	OR (95%CI)	1	0.372 (0.094, 1.471)	0.554 (0.144, 2.135)	2.1 (0.5, 8.821)	0.146
	Ga					
	Range	≤0.2	~0.28	~0.4	>0.4	
	Case/Control	11/18	4/17	6/25	6/7	
	OR (95%CI)	1	0.435 (0.11, 1.719)	0.394 (0.117, 1.332)	0.619 (0.134, 2.861)	0.493
	Ge					
	Range	≤0.33	~0.68	~1.09	>1.09	
	Case/Control	7/9	7/13	9/17	2/30	
	OR (95%CI)	1	1.168 (0.244, 5.582)	0.847 (0.193, 3.706)	0.018 (0.001, 0.257)	0.044
	As					
	Range	≤28.15	~47.17	~72.94	>72.94	
	Case/Control	12/2	7/10	4/26	4/28	
	OR (95%CI)	1	0.167 (0.026, 1.049)	0.027 (0.004, 0.193)	0.026 (0.004, 0.171)	0.002
	Rb					
	Range	≤716.1	~1204.16	~1842.18	>1842.18	
	Case/Control	12/3	9/13	2/26	4/23	
	OR (95%CI)	1	0.171 (0.035, 0.84)	0.025 (0.004, 0.176)	0.044 (0.008, 0.244)	0.002
	Zr					
	Range	≤0.02	~0.05	~0.14	>0.14	
	Case/Control	1/21	6/6	3/15	7/4	
	OR (95%CI)	1	28.198 (2.473, 321.548)	3.315 (0.275, 39.955)	25.585 (2.154, 303.955)	0.037
	Sn					
	Range	≤0.48	~4.49	~10.22	>10.22	
	Case/Control	8/17	6/22	4/22	7/4	
	OR (95%CI)	1	0.547 (0.151, 1.981)	0.197 (0.04, 0.97)	1.616 (0.294, 8.871)	0.146
	Sb					
	Range	≤0.05	~0.13	~0.55	>0.55	
	Case/Control	8/16	9/13	7/13	2/27	
	OR (95%CI)	1	1.583 (0.427, 5.871)	1.116 (0.27, 4.606)	0.108 (0.018, 0.657)	0.061
	Pb					
	Range	≤0.26	~0.76	~1.68	>1.68	
	Case/Control	6/18	3/17	7/20	6/10	
	OR (95%CI)	1	0.373 (0.072, 1.927)	0.78 (0.207, 2.932)	0.873 (0.181, 4.224)	0.681
Meningiomas	Cr					
	Range	≤29.34	~46.98	~104.44	>104.44	
	Case/Control	4/14	2/25	1/19	8/7	
	OR (95%CI)	1	0.258 (0.04, 1.668)	0.165 (0.016, 1.708)	3.316 (0.684, 16.081)	0.053
	Ga					
	Range	≤0.2	~0.28	~0.4	>0.4	
	Case/Control	3/18	0/17	4/25	8/7	
	OR (95%CI)	1	1	0.927 (0.183, 4.703)	5.318 (0.999, 28.302)	0.143
	Ge					
	Range	≤0.33	~0.68	~1.09	>1.09	
	Case/Control	7/9	6/13	2/17	0/30	
	OR (95%CI)	1	1.12 (0.221, 5.691)	0.114 (0.014, 0.94)	1	0.215
	As					
	Range	≤28.15	~47.17	~72.94	>72.94	
	Case/Control	8/2	5/10	2/26	0/28	
	OR (95%CI)	1	0.164 (0.022, 1.247)	0.01 (0.001, 0.151)	1	0.037
	Rb					
	Range	≤716.1	~1204.16	~1842.18	>1842.18	
	Case/Control	8/3	6/13	1/26	0/23	
	OR (95%CI)	1	0.198 (0.036, 1.081)	0.019 (0.002, 0.211)	1	0.032
	Zr					
	Range	≤0.02	~0.05	~0.14	>0.14	
	Case/Control	2/21	4/6	2/15	6/4	
	OR (95%CI)	1	8.094 (1.05, 62.405)	0.935 (0.101, 8.65)	15.602 (2.105, 115.63)	0.026
	Sn					
	Range	≤0.48	~4.49	~10.22	>10.22	
	Case/Control	2/17	1/22	4/22	8/4	
	OR (95%CI)	1	0.257 (0.019, 3.518)	0.894 (0.124, 6.424)	14.303 (1.963, 104.19)	0.021
	Sb					
	Range	≤0.05	~0.13	~0.55	>0.55	
	Case/Control	2/16	7/13	5/13	1/27	
	OR (95%CI)	1	4.396 (0.707, 27.338)	3.33 (0.499, 22.245)	0.248 (0.02, 3.155)	0.082
	Pb					
	Range	≤0.26	~0.76	~1.68	>1.68	
	Case/Control	3/18	5/17	3/20	3/10	
	OR (95%CI)	1	1.7 (0.339, 8.533)	0.688 (0.115, 4.121)	1.584 (0.25, 10.048)	0.702
Cranial and paraspinal nerve tumors	Cr					
	Range	≤29.34	~46.98	~104.44	>104.44	
	Case/Control	1/14	0/25	1/19	5/7	
	OR (95%CI)	1	1	0.715 (0.039, 13.034)	6.696 (0.583, 76.948)	0.496
	Ga					
	Range	≤0.2	~0.28	~0.4	>0.4	
	Case/Control	1/18	0/17	3/25	3/7	
	OR (95%CI)	1	1	1.962 (0.18, 21.409)	3.604 (0.262, 49.632)	0.919
	Ge					
	Range	≤0.33	~0.68	~1.09	>1.09	
	Case/Control	6/9	0/13	1/17	0/30	
	OR (95%CI)	1	1	0.017 (0, 1.01)	1	0.506
	As					
	Range	≤28.15	~47.17	~72.94	>72.94	
	Case/Control	4/2	3/10	0/26	0/28	
	OR (95%CI)	1	3.08 (0.081, 116.495)	1	1	0.947
	Rb					
	Range	≤716.1	~1204.16	~1842.18	>1842.18	
	Case/Control	2/3	1/13	2/26	2/23	
	OR (95%CI)	1	0.049 (0.002, 1.212)	0.063 (0.005, 0.859)	0.042 (0.002, 0.72)	1
	Zr					
	Range	≤0.02	~0.05	~0.14	>0.14	
	Case/Control	0/21	1/6	2/15	2/4	
	OR (95%CI)	1	1	1	1	0.906
	Sn					
	Range	≤0.48	~4.49	~10.22	>10.22	
	Case/Control	0/17	0/22	2/22	5/4	
	OR (95%CI)	1	1	1	1	0.573
	Sb					
	Range	≤0.05	~0.13	~0.55	>0.55	
	Case/Control	1/16	1/13	4/13	1/27	
	OR (95%CI)	1	0.903 (0.038, 21.505)	4.274 (0.359, 50.92)	0.264 (0.012, 5.664)	0.523
	Pb					
	Range	≤0.26	~0.76	~1.68	>1.68	
	Case/Control	1/18	1/17	0/20	4/10	
	OR (95%CI)	1	1.07 (0.058, 19.858)	1	4.471 (0.376, 53.119)	0.841

Metals were calculated in the conditional logistic regression model separately and residential address was tested as confounder. The *p* values were adjusted by FDR.

## Data Availability

The data presented in this study are available on request from the corresponding author due to that the data contains patients and control privacy information.

## Supplementary Materials

The following supporting information can be downloaded at: https://www.mdpi.com/article/10.3390/toxics13020092/s1, Table S1: Tumor volume by type in case group; Table S2: Blood routine examination and routine urine examination of the case group and control group; Table S3: Routine urine examination of the case group and control group; Table S4: Percentage of 47 urinary metals above detection limits. Table S5: Exposure levels of 47 metals in case group and control group; Table S6: Posterior inclusion probability for each metal by BKMR model; Figure S1: Pairwise Pearson correlations among urinary concentrations of 41 metal in the population.
