# Supplementary material for: Association Between Heavy Metal Exposure and Central Nervous System Tumors: A Case-Control Study Using Single and Multi-Metal Models
Source: Toxics. 2025 Jan 26;13(2):92. doi: 10.3390/toxics13020092 (PMC11860643; doi:10.3390/toxics13020092)
Supplement: Supplementary file 1 [file toxics-13-00092-s001.zip › toxics-3397589-supplementary.pdf]

Supplementary Material

For

Association Between Heavy Metal Exposure and Central Nervous System Tumors: A Case-Control Study Using Single and Multi-Metal Models

Sen Luo[1,†], Haixia Wu[2,†], Fang Xiao[3], Tianwen Yang[4], Wei Wang[4], Hang Du[4,*], Peng Su[1,*]

[1] Department of Occupational and Environmental Health, School of Public Health, Chongqing Medical University, Chongqing, 400016, People's Republic of China.

[2] Nursing Department of the First Affiliated Hospital of Chongqing Medical University, Chongqing , People's Republic of China.

[3] Orthopedics Department of Sichuan Provincial People's Hospital, Chengdu, 610072, People's Republic of China.

[4] Chongqing Key Laboratory of Prevention and Treatment for Occupational Diseases and Poisoning, The First Affiliated Hospital of Chongqing Medical and pharmaceutical College, Chongqing , People's Republic of China.

[†]Authors contributed equally to this work

***Corresponding author**

Hang Du, Email: 646698773@qq.com

Peng Su, E-mail: 103335@cqmu.edu.cn

Contents of supplementary materials

Table S1 Tumor volume by type in case group.

Table S2 Blood routine examination and routine urine examination of the case group and control group.

Table S3 Routine urine examination of the case group and control group.

Table S4 Percentage of 47 urinary metals above detection limits

Table S5 Exposure levels of 47 metals in case group and control group.

Table S6 Posterior inclusion probability for each metal by BKMR model.

Figure S1 Pairwise Pearson correlations among urinary concentrations of 41 metal in the population .

Table S1

Tumor volume by type in case group

Classification	Quartile 1	Quartile 2	Quartile 3	Quartile 4
Gliomas n (%)	3 (10.7)	2 (7.1)	5 (17.9)	6 (21.4)
Meningiomas n (%)	3 (20)	3 (20)	4 (26.7)	3 (20)
Cranial and paraspinal nerve tumors n (%)	1 (14.3)	2 (28.6)	0 (0)	2 (28.6)
Tumors of the sellar region n (%)	4 (80)	0 (0)	1 (20)	0 (0)
Mesenchymal, non-meningothelial tumors n (%)	0 (0)	1 (50)	0 (0)	0 (0)
Neuronal tumors n (%)	0 (0)	1 (50)	1 (50)	0 (0)
Germ cell tumors n (%)	0 (0)	2 (100)	0 (0)	0 (0)
Ependymal tumors n (%)	0 (0)	1 (100)	0 (0)	0 (0)
Embryonal tumors n (%)	0 (0)	0 (0)	1 (100)	0 (0)

Table S2

Blood routine examination and routine urine examination of the case group and control group

Parameter	Case	Control	*p* value
Prealbumin (mg/L) Mean ±SD	251.17±75.018	283.4±49.024	0.149
Total protein (g/L) Median(P25,P75)	68(63,74)	76(74,78.75)	＜0.001
Albumin (g/L) Median(P25,P75)	42(37,46)	45(43,46)	0.001
Globulin (g/L) Median(P25,P75)	23.5(21.75,27)	31(29,34)	＜0.001
Albumin to globulin ratio Median(P25,P75)	1.95(1.7,2.1)	1.4(1.3,1.5)	＜0.001
Total bilirubin (μmol/L) Median(P25,P75)	9.7(6.625,12.325)	13.6(11.45,17.65)	＜0.001
Direct bilirubin (μmol/L) Median(P25,P75)	3.3(2.5,4.275)	4.6(4,5.55)	＜0.001
Alanine aminotransferase (u/L) Median(P25,P75)	19(14,37)	18(13,32.75)	0.601
Aspartate aminotransferase (U/L) Median(P25,P75)	22(18,27)	20(16,25)	0.227
Alkaline phosphatase (U/L) Median(P25,P75)	74(58.5,89)	73(65.75,90.75)	0.513
Gamma glutamyl transferase (U/L) Median(P25,P75)	20(14,28.25)	26(18,38)	0.025
Lactate dehydrogenase (U/L) Median(P25,P75)	154.5(136.75,181.25)	167(151,216)	0.157
Blood urea (mmol/L) Median(P25,P75)	5.05(3.775,6.075)	5(4.3,6)	0.419
Blood creatinine (μmol/L) Median(P25,P75)	62(51,74)	75(61,84)	＜0.001

Blood uric acid (μmol/L) Median(P25,P75)	249(181.5,319.5)	401(330,448)	＜0.001
Total cholesterol (mmol/L) Median(P25,P75)	4.83(3.74,5.3225)	5.205(4.6625,5.8325)	0.030
Triglyceride (mmol/L) Median(P25,P75)	1.325(0.985,2.05)	1.24(0.845,1.765)	0.629
High-density lipoprotein cholesterol (mmol/L) Median(P25,P75)	1.31(1.1475,1.5725)	1.395(1.2075,1.61)	0.366
Low-Density lipoprotein cholesterol (mmol/L) Mean ±SD	2.878±0.716	2.771±0.685	0.604
Blood glucose (mmol/L) Median(P25,P75)	5.3(4.9,6.25)	5.2(4.9,5.4)	0.205
Leucocyte (10^9/L) Median(P25,P75)	6.575(5.01,9.7525)	5.73(4.91,6.76)	0.010
Erythrocyte (10^12/L) Mean ±SD	4.368±0.661	4.884±0.479	＜0.001
Hemoglobin (g/L) Median(P25,P75)	132(113.5,147)	151(135,159)	＜0.001
Hematocrit (%) Mean ±SD	39.515±6.065	44.302±4.964	＜0.001
Mean corpuscular volume (fl) Median(P25,P75)	91(86.65,93.95)	90(87.9,93.6)	0.665
Mean corpuscular hemoglobin (pg) Median(P25,P75)	29.5(28.7,31.3)	30.5(29.6,31.6)	0.007
Mean corpuscular hemoglobin concentration (g/L) Median(P25,P75)	325(318,336)	338(332,341)	＜0.001
Blood platelet (10^9/L) Median(P25,P75)	243.5(207,312)	236(202,276)	0.294
Platelet distribution width (fl) Median(P25,P75)	12.25(10.675,14.3)	16.3(16.1,16.5)	＜0.001
Mean platelet volume (fl) Median(P25,P75)	10.5(9.9,11.5)	10.4(9.8,11.1)	0.275
Plateletcrit (%) Median(P25,P75)	0.26(0.23,0.3125)	0.25(0.22,0.29)	0.069

Neutrophil percentage (%) Median(P25,P75)	63.25(55.175,75.65)	57.95(51.825,62.625)	＜0.001
Lymphocyte percentage (%) Median(P25,P75)	26.75(15.8,34.65)	32.7(28.475,37.475)	＜0.001
Monocyte percentage (%) Median(P25,P75)	7.05(5.95,8.325)	5.8(4.9,7)	＜0.001
Eosinophil percentage (%) Median(P25,P75)	1.45(0.275,2.3)	2.4(1.2,3.6)	0.001
Basophil percentage (%) Median(P25,P75)	0.4(0.2,0.6)	0.5(0.3,0.7)	0.020
Neutrophil (10^9/L) Median(P25,P75)	3.9(2.955,6.575)	3.28(2.52,4.09)	0.004
Lymphocyte (10^9/L) Mean±SD	1.614±0.52	1.863±0.582	0.018
Monocyte (10^9/L) Median(P25,P75)	0.45(0.35,0.6)	0.33(0.2675,0.415)	＜0.001
Eosinophil (10^9/L) Median(P25,P75)	0.09(0.03,0.17)	0.13(0.08,0.18)	0.026
Basophil granulocytes (10^9/L) Median(P25,P75)	0.03(0.02,0.03)	0.03(0.02,0.04)	0.358
Red blood cell distribution width Median(P25,P75)	12.85(12.325,13.65)	13(12.6,13.35)	0.913
Specific gravity Median(P25,P75)	1.02(1.014,1.026)	1.021(1.017,1.025)	0.386

Table S3

Routine urine examination of the case group and control group

Parameter	Case		Control	*p* value
Urine PH n(%)	5	3(4.8)	15(21.1)	＜0.001
	5.5	11(17.5)	17(23.9)	
	6	8(12.7)	15(21.1)	
	6.5	14(22.2)	7(9.9)	
	7	10(15.9)	5(7)	
	7.5	9(14.3)		
Urine Leukocytes n(%)	negative	48(77.4)	50(70.4)	0.698
	positive	7(11.3)	9(12.7)	
Urine nitrite n(%)	negative	52(83.9)	59(83.1)	
	positive	3(4.8)		
Protein urine n(%)	negative	53(85.5)	59(83.1)	0.019
	positive	2(3.2)	12(16.9)	
Urine glucose n(%)	negative	50(79.4)	56(78.9)	0.479
	positive	5(87.9)	3(4.2)	

Urine ketones n(%)	negative	55(87.3)	59(83.1)	
	positive		12(16.9)	
Urine occult blood n(%)	negative	42(66.7)	41(57.7)	0.410
	positive	13(20.6)	18(25.4)	
Urobilinogen n(%)	negative	54(85.7)	57(80.3)	
	positive	1(1.6)	2(2.8)	
Urine bilirubin n(%)	negative	55(87.3)	59(93.1)	
	positive			
Urine conductivity n(%)	Rank1	1(1.6)		＜0.001
	Rank2	29(46)	10(14.1)	
	Rank3	18(28.6)	38(53.5)	
	Rank4	4(6.3)	5(7)	

Table S4

Percentage of 47 urinary metals above detection limits

	LOD	Case n(%)	Controls n(%)	*p* value
V	0.1494	62(98.4)	66(93)	0.068
Cr	0.0522	63(100)	65(91.5)	0.007
Mn	0.1275	59(93.7)	63(88.7)	0.160
Fe	8.3442	59(93.7)	66(93)	0.490
Co	0.0015	60(95.2)	67(94.4)	0.419
Ni	0.0153	58(92.1)	69(97.2)	0.733
Cu	0.9378	50(79.4)	66(93)	0.070
Zn	0.4644	61(96.8)	66(93)	0.133
Ga	0.0027	62(98.4)	67(94.4)	0.081
Ge	0.0066	59(93.7)	69(97.2)	0.830
As	0.1071	62(98.4)	66(93)	0.068
Rb	0.0213	62(98.4)	65(91.5)	0.037
Sr	0.0444	63(100)	65(91.5)	0.007
Y	0.0006	38(60.3)	10(14.1)	＜0.001
Zr	0.0024	47(74.6)	46(64.8)	0.147
Nb	0.0609	9(14.3)	4(5.6)	0.082
Mo	0.0171	63(100)	65(91.5)	0.007
Rh	0.0156	45(71.4)	55(77.5)	0.606
Pd	0.0009	32(50.8)	15(21.1)	＜0.001
Ag	0.0030	2(3.2)	46(64.8)	＜0.001
Cd	0.0006	62(98.4)	65(91.5)	0.037
In	0.0015	40(63.5)	67(94.4)	＜0.001
Sn	0.0120	60(95.2)	65(91.5)	0.186
Sb	0.0027	60(95.2)	70(98.6)	1
Cs	0.0001	62(98.4)	66(93)	0.068
Ba	0.0315	62(98.4)	58(81.7)	0.001
La	0.0009	45(71.4)	16(22.5)	＜0.001
Ce	0.0009	41(65.1)	16(22.5)	＜0.001
Pr	0.0001	18(28.6)	31(43.7)	0.092
Nd	0.0001	40(63.5)	7(9.9)	＜0.001
Sm	0.0001	18(28.6)	23(32.4)	0.710
Eu	0.0001	18(28.6)	0(0)	＜0.001
Gd	0.0033	63(100)	13(18.3)	＜0.001
Tb	0.0001	35(55.6)	42(59.2)	0.816
Dy	0.0001	22(34.9)	8(11.3)	0.001
Ho	0.0001	61(96.8)	0(0)	＜0.001
Er	0.0006	49(77.8)	4(5.6)	＜0.001
Tm	0.0001	31(49.2)	1(1.4)	＜0.001
W	0.0009	58(92.1)	67(94.4)	0.952
Re	0.0001	49(77.8)	65(91.5)	0.075
Pt	0.0001	28(44.4)	1(1.4)	＜0.001
Au	0.0006	6(9.5)	4(5.6)	0.513
Hg	0.0801	47(74.6)	67(94.4)	0.007
Tl	0.0001	63(100)	66(93)	0.015

Pb	0.0318	54(85.7)	65(91.5)	0.559
Bi	0.0066	37(58.7)	58(81.7)	0.009
U	0.0001	50(79.4)	39(54.9)	0.002

Table S5

Exposure levels of 47 metals in case group and control group

	Case		Control		Adjusted
	N	Median (P25, P75)	N	Median (P25, P75)	*p* value
V	62	34.345(20.726,49.513)	66	55.88(41.63,67.465)	<0.001
Cr	63	88.838(43.133,138.998)	65	59.64(49.94,74.39)	0.050
Mn	59	2.787(1.247,4.087)	63	5.02(3.36,6.56)	<0.001
Fe	59	464.88(277.367,712.54)	66	801.97(565.865,1204.76)	<0.001
Co	60	0.363(0.248,0.533)	67	0.53(0.35,0.75)	0.003
Ni	58	3.543(2.089,4.628)	69	3.9(2.37,5.3)	0.455
Cu	50	8.912(5.693,15.248)	66	12.015(7.665,18.395)	0.066
Zn	61	270.389(148.41,394.364)	66	336.275(205.68,475.73)	0.198
Ga	62	0.317(0.208,0.48)	67	0.26(0.2,0.34)	0.066
Ge	59	0.429(0.247,0.733)	69	0.96(0.64,1.59)	<0.001
As	62	28.277(18.912,40.2)	66	68.8(49.975,87.785)	<0.001
Rb	62	759.098(492.815,1211.368)	65	1689.76(1190.26,2491.57)	<0.001
Sr	63	98.353(50.96,153.98)	65	154.9(100.86,222.09)	<0.001
Y	38	0.022(0.015,0.048)	10	0.02(0.02,0.025)	0.743
Zr	47	0.08(0.035,0.227)	46	0.035(0.015,0.06)	<0.001
Nb	9	0.073(0.013,0.228)	4	0.595(0.095,1.56)	0.170
Mo	63	19.782(3.287,39.253)	65	55.135(38.185,92.275)	<0.001
Rh	45	0.12(0.07,0.167)	55	0.405(0.225,0.725)	<0.001
Pd	32	0.037(0.015,0.068)	15	0.005(0.005,0.025)	<0.001
Ag	2		46	0.005(0.005,0.005)	
Cd	62	0.863(0.485,2.025)	65	1.075(0.665,1.955)	0.136
In	40	0.05(0.033,0.092)	67	0.165(0.105,0.265)	<0.001
Sn	60	8.304(0.566,17.885)	65	3.425(0.465,6.465)	0.001

Sb	60	0.1(0.053,0.193)	70	0.385(0.06,0.815)	0.017
Cs	62	3.35(2.04,5.744)	66	6.79(5.095,9.13)	<0.001
Ba	62	3.995(1.528,8.985)	58	1.01(0.405,2.47)	<0.001
La	45	0.033(0.011,0.203)	16	0.075(0.04,0.075)	0.463
Ce	41	0.029(0.01,0.067)	16	0.02(0.02,0.055)	0.796
Pr	18	0.02(0.007,0.02)	31	0.02(0.02,0.02)	0.062
Nd	40	0.02(0.007,0.027)	7	0.015(0.015,0.055)	0.730
Sm	18	0.02(0.013,0.02)	23	0.015(0.015,0.015)	0.064
Eu	18	0.02(0.013,0.02)	0	0(0,0)	
Gd	63	151.26(37.36,568.87)	13	0.015(0.015,0.015)	<0.001
Tb	35	0.02(0.013,0.04)	42	0.015(0.015,0.015)	0.011
Dy	22	0.013(0.013,0.02)	8	0.02(0.02,0.02)	0.010
Tm	61	2.82(0.5,9.273)	0	0(0,0)	
Er	49	0.1(0.02,0.293)	4	0.02(0.02,0.02)	0.093
Hf	31	0.013(0.007,0.02)	1	0.015(0.015,0.015)	0.824
W	58	0.293(0.135,0.503)	67	0.675(0.475,1.075)	<0.001
Re	49	0.04(0.02,0.1)	65	0.1(0.06,0.18)	<0.001
Pt	28	0.04(0.02,0.248)	1	0.095(0.095,0.095)	0.733
Au	6	0.013(0.013,0.013)	4	0.025(0.015,0.05)	0.011
Hg	47	0.602(0.28,1.062)	67	0.4(0.24,0.62)	0.050
Tl	63	0.24(0.153,0.48)	66	0.52(0.375,0.7)	<0.001
Pb	54	0.893(0.34,1.858)	65	0.66(0.24,1.3)	0.215
Bi	37	0.113(0.029,0.46)	58	0.03(0.01,0.095)	0.003
U	50	0.02(0.02,0.043)	39	0.02(0.02,0.02)	0.010

Table S6

Posterior inclusion probability for each metal by BKMR model

	PIPs
Cr	0.9226
Ga	0.9684
Ge	0.9418
As	0.9024
Rb	0.7952
Zr	0.7704
Sn	0.9904
Sb	1.0000
Pb	0.9784

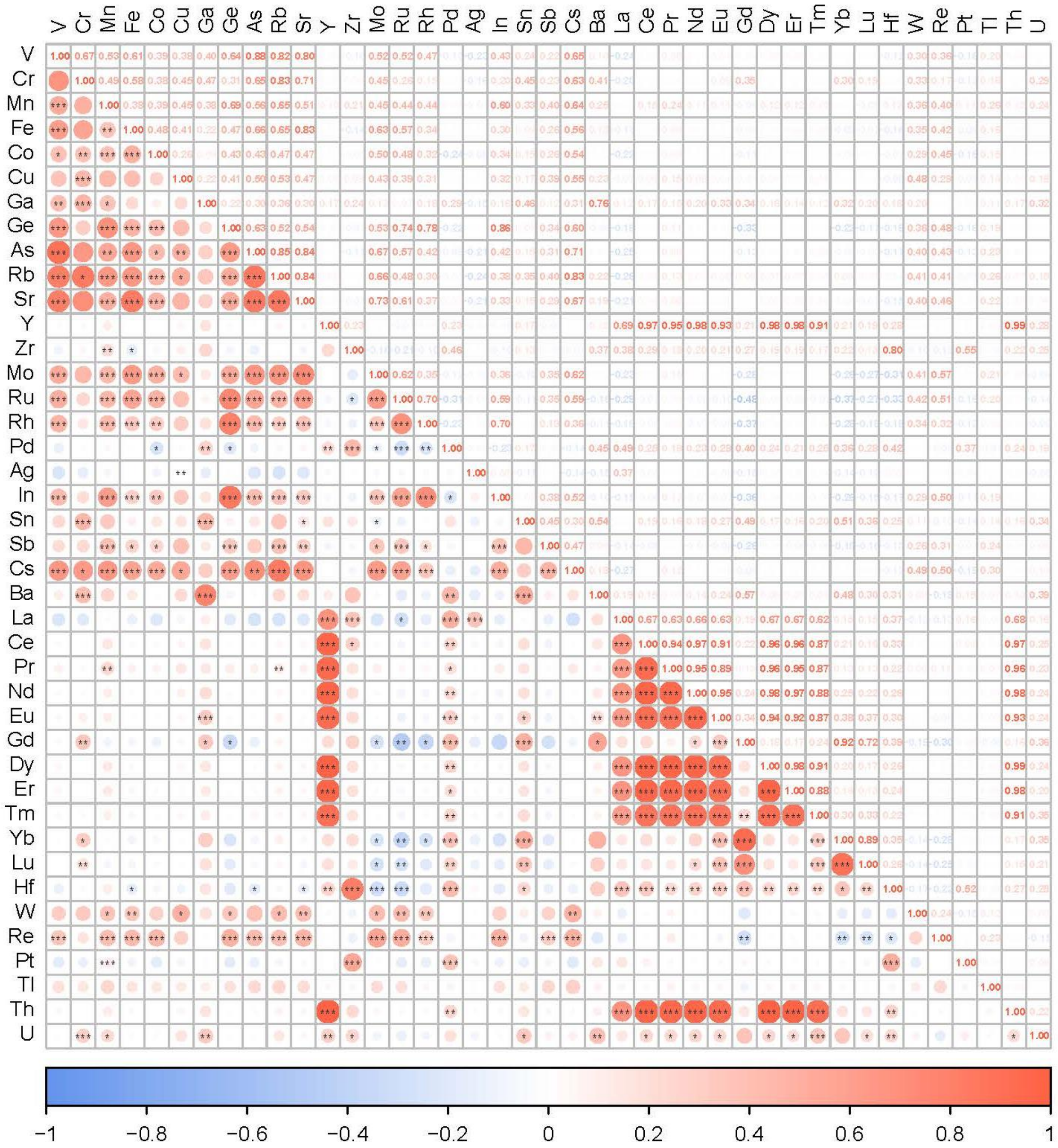

Fig. S1. Pairwise Pearson correlations among urinary concentrations of 41 metal in the population .

When r ≥ 0.7, it is considered that there is a positive correlation between two metals, and the sources of the two metals are the same
